# Individualized stress detection using an unmodified car steering wheel

**DOI:** 10.1038/s41598-021-00062-7

**Published:** 2021-10-19

**Authors:** Stephanie Balters, Nikhil Gowda, Francisco Ordonez, Pablo E. Paredes

**Affiliations:** 1 Department of Psychiatry and Behavioral Science, Stanford University, Stanford, CA USA; 2 Alliance Innovation Lab Silicon Valley, Santa Clara, CA USA; 3 Computer Science Department, Universidad San Francisco de Quito, Quito, Ecuador; 4 Department of Epidemiology and Population Health, Stanford University, Stanford, CA USA

**Keywords:** Risk factors, Signs and symptoms, Engineering

## Abstract

In-car passive stress sensing could enable the monitoring of stress biomarkers while driving and reach millions of commuters daily (i.e., 123 million daily commuters in the US alone). Here, we present a nonintrusive method to detect stress solely from steering angle data of a regular car. The method uses inverse filtering to convert angular movement data into a biomechanical Mass Spring Damper model of the arm and extracts its damped natural frequency as an approximation of muscle stiffness, which in turn reflects stress. We ran a within-subject study (*N* = 22), in which commuters drove a vehicle around a closed circuit in both stress and calm conditions. As hypothesized, cohort analysis revealed a significantly higher damped natural frequency for the stress condition (*P* = .023, *d* = 0.723). Subsequent automation of the method achieved rapid (i.e., within 8 turns) stress detection in the individual with a detection accuracy of 77%.

## Introduction

In 2007, the American Psychological Association started to flag stress as a major health problem in the US^1^. The report showed that one-third of Americans were living with extreme stress and nearly half of Americans (48%) believed that their stress had increased over the past 5 years. The numbers have increased even further since^2,3^, and recently the COVID-19 pandemic has transformed stress into a national health crisis^4–7^. The adverse effects of repetitive acute stress and chronic stress can have physical, behavioural and/or neuropsychiatric manifestations^8–10^. The World Economic Forum and the Lancet Commission on Global Mental Health propose the advancement of digital technology, to both understand the early (prodromal) effects of environmental factors such as workplace stress and to advance interventions for the pre-disease (subsyndromal) stages of mental illness^11^. Advocates of Precision Health argue that we can intercept risk factors of disease by regular measurements of biomarkers^12–16^. Passive sensors are an important application feature that enable frequent monitoring of health states without requiring designated changes in user behaviour^12^. Among the various spaces and scenarios where such smart sensing can occur on a frequent basis, the car can become a valuable source of data^17^. Given that 123 million people in the US alone drive their own car to work^18^, an in-car stress sensing system could monitor about 87% of the US workforce and measure biomarkers of stress twice a day, every workday.

**Figure 1:**
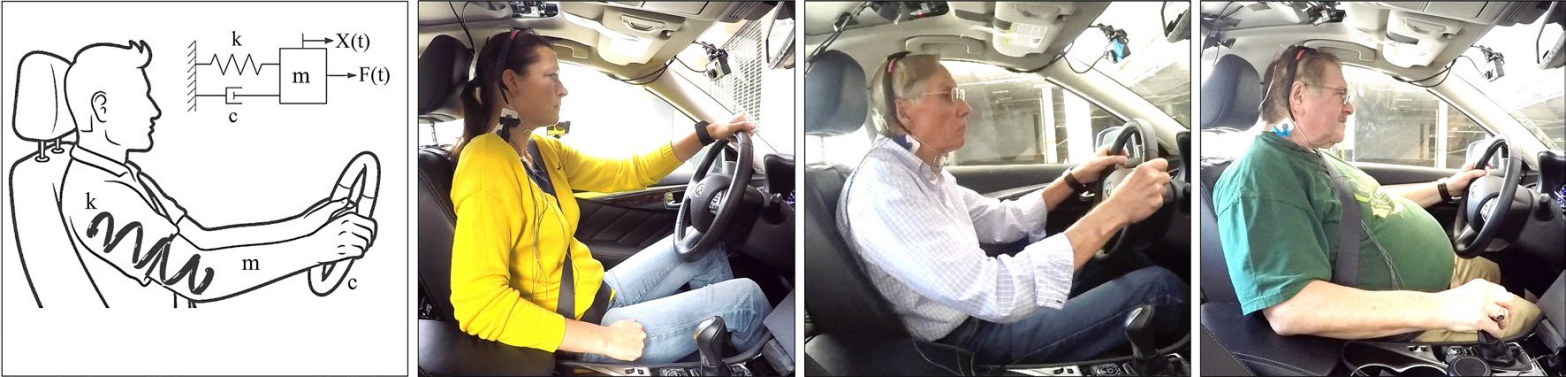
Individualized driver stress detection method using an unmodified car steering wheel.

Early concepts of passive sensors tested in automotive simulator environments have shown promise to measure vital signs related to stress, such as heart rate and breathing rate assessments through Ultra Wide Band radar and WiFi signals^19,20^. Integration of these concepts into the moving vehicle is still an outstanding engineering challenge^19,20^ and the additional hardware cost is a hindarance. In contrast to such concepts that require additional sensor equipment, we introduce a novel “sensor-less”^21^ in-car stress sensing method to passively detect stress purely from steering wheel data. In modern passenger vehicles, these data are readily available on the vehicle’s communication network. The proposed steering wheel stress sensing method is based on the psychophysiological mechanism that stress has a direct effect on musculoskeletal activity mediated by the somatic nervous system. Muscle tension in the neck, shoulders, and arms increases with stress and anxiety^22^, even at physiological rest (i.e., without limb movement)^23,24^. Traditional laboratory stressors, such as mental arithmetic, have shown effects on the shoulder’s trapezius muscle, as demonstrated with electromyography (EMG) measures^25^. Crucially, the trapezius muscle is active during arm movements^26^, such as steering activity^27^. Researchers have indirectly assessed changes in muscle stiffness during everyday activities like clicking on a computer mouse^28^, typing on a keyboard^29^ or using a trackpad^30^. Sun et al.^31^ were the first to show a correlation between stress level and arm-induced movement of using a computer mouse. In the present study, we adapted the movement-based approach from Sun et al.^31^ to a steering wheel in a moving car. Specifically, we applied an inverse filtering technique, called linear predictive coding (LPC)^32^, on the steering angle data, to approximate a biomechanical Mass Spring Damper (MSD) model and used the damped natural frequency of the MSD as estimate for muscle tension (Fig. 1). This nonintrusive sensing approach is promising for future adoption by the automotive industry, as car data could be used to detect a driver’s stress levels at no additional hardware cost. Before conducting the present study in a moving vehicle, we generated pilot data within a simulator environment^33^. The related findings provided an early proof-of-concept of the proposed steering wheel stress sensing method. Working with a real car, however, presents additional technical and methodological challenges. For example, the steering angle data collected from a car’s controller area network (CAN) bus have a considerably lower sampling frequency and lower precision than a simulator steering wheel. Furthermore, in real world testing, multiple variables such as driving forces, road vibrations, tire dynamics, wheel mass inertia, and steering support torque are additional factors that can affect the biomechanical model of a driver’s arm. We therefore set out to address our first research question:

- ***RQ1:*** Can we detect driver stress through steering wheel angle data in a moving car using an LPC-based biomechanical model?

After successful a validation of the steering stress sensing method on cohort level, we transitioned into idiosyncratic analysis. Specifically, we aimed to develop an automated version of the stress sensing method that can detect stress in the individual. Our second research question is as follows:

- ***RQ2:*** Can we automate the LPC-based steering wheel stress sensing method to detect stress in individual drivers?

To answer these questions, we ran a within-subject experiment with frequent commuters (*N* = 24) driving an unmodified passenger car in a closed course. We collected CAN bus steering angle data during a stressful driving condition and a calm driving condition. Additionally, we captured subjective stress assessments and physiological measures (i.e., heart rate and heart rate variability) to validate stress responses; and measured ancillary driving behaviour data (e.g., average speed, acceleration) to control for driving behaviour that could potentially affect steering behaviour. We hypothesized that the LPC-based steering wheel stress sensing method enabled accurate detection of stress between the two conditions, both across the cohort and in the individual driver.

## Results

### Cohort analysis

#### Stress stimulus validation

Prior to evaluating the effectiveness of the proposed stress sensing method, we analysed both the self-reported stress assessments (i.e., perceived level of *stress*, *arousal*, *valence*, and *tension*) and the physiological measures (i.e., *heart rate* and *heart rate variability*) to validate that the applied calm and stress pre-driving stimuli worked as intended and participants were more stressed during stress exposure compared to calm conditions. We expected perceived *stress*, *arousal*, *valence*, and *tension* and physiological *heart rate* (*HR*) measures to increase with stress, and *heart rate variability* (*HRV*) to decrease with stress. Specifically, we tested for differences between: (1) CALM STIMULUS and STRESS STIMULUS to test whether the stimuli worked; (2) CALM DRIVE and STRESS DRIVE to test whether the effects of the two stimuli were maintained while driving; (3) CALM STIMULUS and CALM DRIVE and (4) STRESS STIMULUS and STRESS DRIVE to test whether there were differences in stress induced by the pre-drive stimulus compared to the subsequent driving task. We min–max normalized self-reported and physiological data and corrected against baseline. In the next step, we used permutation tests for statistical analysis. First, we ran an original Student’s t-test to obtain the t-statistic of the data across the population in Matlab 2021a (MathWorks, https://www.mathworks.com/products/matlab.html). We then randomly permuted each group of values (per user) across all users. We generated 100,000 permutations and computed the t-statistic for each group. The observed t-statistic can be considered a random sample from the permutation distribution. We further counted how often a larger (or smaller) t-statistic was observed in contrast to the original data and divided that by the number of permutations performed. In this case, “*P*” is the probability of finding a test statistic for the group comparison at least as high (or low) as our original data^34^ (not to be confused with *p* values from traditional parametric tests). We assigned significance if a value was higher than 95% or lower than 5% percent results, and reported significance accordingly (1 – *P*; *P*). Bonferroni correction was used for multi-comparison.

**Figure 2:**
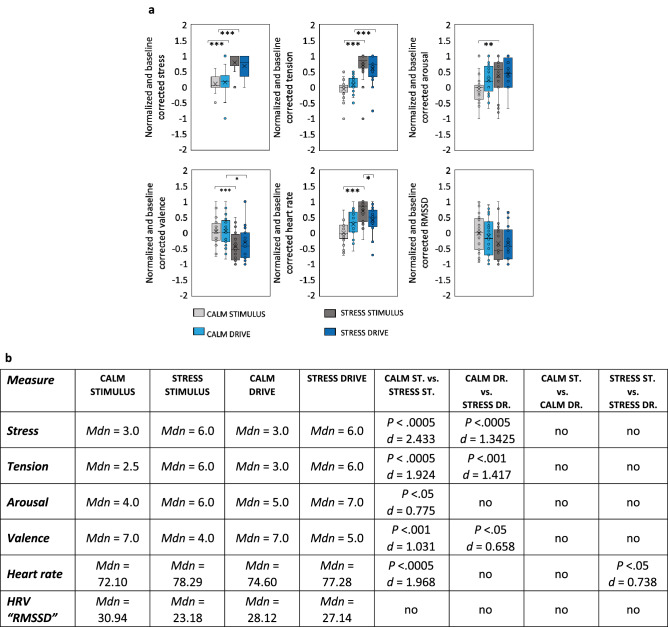
Results of the stressor validation analysis to establish a ground-truth assessment of whether participants were more stressed during stress exposure compared to calm conditions. (**a**) Boxplots of normalized and baseline corrected subjective and psychophysiological metrics. Permutation probability *P *values are corrected for multiple-comparisons with ***P < 0.001; **P < 0.01, and *P < 0.05. (**b**) Permutation results with adjusted probability *p* values after Bonferroni correction, and Cohen’s d as effect size measure reference.

Boxplots and results of statistical tests are depicted in Fig. 2. For perceived *stress* and *tension*, the mean values were significantly higher during STRESS STIMULUS compared to CALM STIMULUS as well as during STRESS DRIVE compared to CALM DRIVE. As expected, self-reported stress and tension levels were significantly higher in the presence of the stress stimulus and were maintained during the driving task as we did not observe significant decreases between STRESS STIMULUS and STRESS DRIVING. Pearson correlation analysis of the original values across all four conditions revealed high correlation between stress and tension with *r* = 0.869, *p* < 0.0001. *Arousal* was higher for STRESS STIMULUS versus CALM STIMULUS, but interestingly, there was no difference between the STRESS DRIVING and CALM DRIVING conditions. This lack of observed differentiation is perhaps because the arousal perceived due to driving itself is not perceived as stress. *Valence* results showed a significant decrease in CALM STIMULUS versus in STRESS STIMULUS and we observed similar results in CALM DRIVE versus STRESS DRIVE. These findings indicate that participants experienced a negatively valenced stress response (i.e., distress) during the stressful conditions. Overall, we observed large effect sizes (Cohen’s d) across the subjective measures, and we could confirm that the stress stimulus worked as expected—participants were more stressed during STRESS DRIVE than during CALM DRIVE. There was a reduction in the effect of the initial stimulus as participants carried on with driving. *Note:* Two participants did not report increased levels of perceived stress and muscle tension and are therefore excluded from analysis of RQ1 and RQ2 (i.e., resulting in *N* = 22).

The *HR* permutation test showed that STRESS STIMULUS was higher than CALM STIMULUS and STRESS DRIVE. Hence, the stressor elicited a physiological stress which decreased as the drive progressed. Similarly, the *HRV* measure “Root Mean Square of Successive Differences (RMSSD) between heartbeats” showed the expected tendencies of decreased values in the presence of stress; however, after Bonferroni correction, significance thresholds were not reached (*P* = 0.061). The overall results validate that the experimental stimuli were effective; individuals were experiencing more stress during the stress conditions compared to the calm conditions.

#### Normal driving behaviour validation

As the last validation step, we verified that the experimental setup did not have an impact on driving behaviour, which could contaminate the stress signal in the steering angle data. For example, a participant could drive faster during the STRESS DRIVE condition, which in turn, would influence the steering input due to increased longitudinal and/or lateral forces. Because both forces are a function of speed^35^, we validated that there were no differences in the metrics *speed during turn segments* (mph) and *duration of turn segments* (sec) between the two conditions. We extracted speed data from the vehicle’s CAN bus, sampled at 50 Hz. As described below, we tested for two driving durations (i.e., ENTIRE DRIVE and INITIAL 8 TURNS). Permutation tests for neither duration revealed significant differences for any of the driving dynamics metrics (Table S1 in the supplementary material). These findings suggest that there were no differences in driving behaviour between CALM DRIVE and STRESS DRIVE, and therefore, our stimuli did not elicit a signal other than steering behaviour. As a result, we were able to test our experimental hypotheses.

### LPC-based steering wheel stress sensing method (RQ1)

#### Experimental hypotheses

We tested the stress sensing method for the entire duration (i.e., ENTIRE DRIVE) of the two driving conditions as well for a shorter duration (i.e., INITIAL 8 TURNS). The rationale for the shorter duration was that we aimed to develop a *rapid* stress sensing method which could enable just-in-time stress management interventions during the commute (e.g., in-car slow breathing interventions^36,37^). Our pilot study in the simulator determined that a minimum of eight turns was needed to find a difference in damped natural frequency across stress and calm conditions^33^. Specifically, we formulated following experimental hypotheses:

- ***H1:*** LPC-modelled *damped natural frequency* (*ω*) is higher during the STRESS DRIVE compared to the CALM DRIVE for the ENTIRE DRIVE.
- ***H2:*** LPC-modelled *damped natural frequency* (*ω*) is higher during the STRESS DRIVE compared to the CALM DRIVE for the INITIAL 8 TURNS.

#### LPC model fit validation

**Figure 3:**
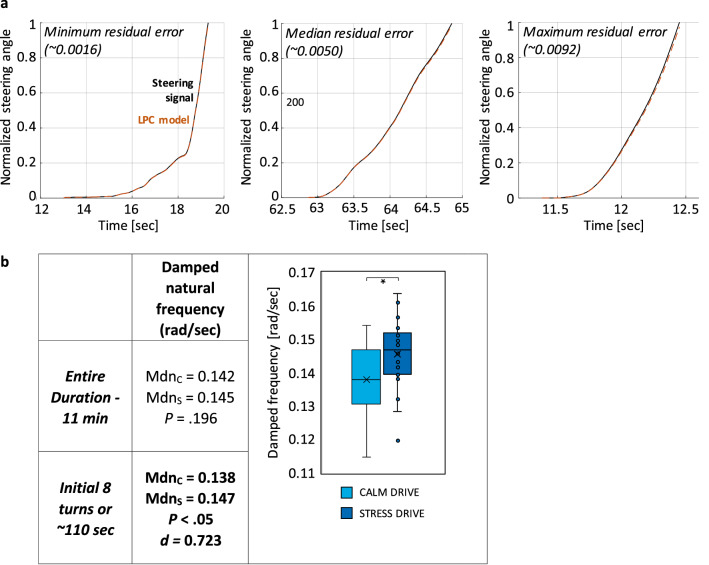
Results for cohort analyses. (**a**) LPC model fit with minimum, median, maximum variance of the residual errors across participants. Overall LPC models showing good quality of fit in all three examples. (**b**) Shows median values and boxplots of damping frequencies, with “c” = calm drive, “s” = stress drive, and ‘d” = Cohen’s d. *P* values need to fall below 0.0125 to be significant due to Bonferroni correction for multi-comparison.

Prior to testing our hypotheses, we evaluated the quality of the LPC model fit by inspecting the variance of the residual errors on the ENTIRE DRIVE, as well as the INITIAL 8 TURNS which represents the initial 110 s of driving. We calculated Tukey Anomaly outlier thresholds for STRESS DRIVE and CALM DRIVE for both the ENTIRE DRIVE and INITIAL 8 TURNS and used the minimum of the four values as overall LPC model fit threshold (i.e., fitting threshold = 0.0093) (Fig. S1 in the supplementary material). This outlier correction method eliminated 7.8% of ENTIRE DRIVE data and 8.2% of INITIAL 8 TURNS data. Overall, we observed a median of the variance of the residual error of 0.0050, with minimum values around 0.0016 and maximum values 0.0093 (Fig. 3a). The LPC model error variance was overall low^38,39^. While examining if there were differences across the driving conditions, permutation tests revealed no differences between CALM DRIVE and STRESS DRIVE for INITIAL 8 TURNS (*P* = 0.143); however, for ENTIRE DRIVE, the LPC variance of the residual error was higher during CALM DRIVE than STRESS DRIVE (*P* = 0.006; with low effect size *d* = 0.125). To assure that this difference in LPC model fit did not impact further analyses, we executed a correlation analysis between residual errors and damped natural frequency values. Results showed no correlation between the two metrics (*P* = 0.290), and small differences across conditions shall not affect analysis moving forward.

#### Experimental hypothesis testing

In the first step, we tested for the ENTIRE DRIVE of 10 laps in both stress conditions (*M*_CALM DRIVE_ = 11.9 min, *SD* = 1.9; *M*_STRESS DRIVE_ = 11.6 min, *SD* = 2.1). During CALM DRIVE we obtained (*M* = 62 turn segments, *SD* = 9) per participant and for the STRESS DRIVE condition we obtained (*M* = 60 turn segments, *SD* = 9) per participant. A permutation test revealed no difference between in damped natural frequencies *ω*_CALM DRIVE_ and *ω*_STRESS DRIVE_. During the INITIAL 8 TURNS there was an average of (*M* = 8.6 turn segments, *SD* = 2.2) per participant during CALM DRIVE and (*M* = 9.8 turn segments, *SD* = 2.2) per participant during STRESS DRIVE. Permutation test revealed a significant difference between *ω*_CALM DRIVE_ and *ω*_STRESS DRIVE_, with a strong effect size (*Cohen’s d* = 0.723) (Fig. 3b). These results confirm our experimental hypothesis of a significantly higher damped natural frequency in stressed driving conditions for the data segment of the INITIAL 8 TURNS.

### Individualized steering wheel stress sensing (RQ2)

**Figure 4:**
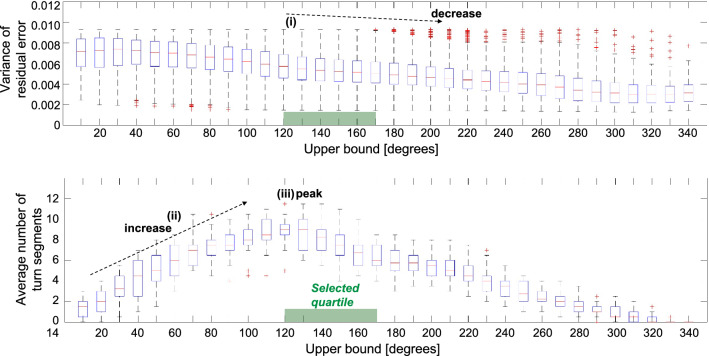
*Variance of residual error* and *average number of turn segments* across the iterated *upper bound* segmentation parameters, with decreasing variance of residual error (i) and increasing average number of turn segments (ii) across *upper bound* values. The peak in average number of turn segments is at 120° *upper bound* (iii). Please find the definition of *upper bound* in the *Methods* section.

After validating the steering wheel stress sensing method across cohort, our next aim was to develop an automatic (data-driven) turn segmentation process that provides automated stress assessment in the individual. We developed the process based on cohort data and applied the process to individual data thereafter. Because we aimed at generating a rapid sensing method, we used the INITIAL 8 TURNS cohort data of the previous section (which had been corrected for outliers, i.e., fitting threshold < 0.0093). Prior to inspecting the cohort data, we defined three design considerations for the automatic turn segmentation process: (1) good quality of LPC model fit (i.e., low variance of residual error values); (2) multiple curve segments to have sufficient input data for the stress sensing method (e.g., eight turn segments); (3) assessment of the damped natural frequency values over a range of *upper bound values* (in contrast to using a single *upper bound value*) to avoid Type II errors. To generate an automated turn segmentation process, we inspected the *variance of residual error* and *average number of turn segments* across the iterated *upper bond* segmentation parameters (Fig. 4). We observed that the *variance of residual error* decreased with an increase in *upper bound values* (Fig. 4i). This relation is expected since longer turn segments include more data, and hence model fit is better. Additionally, we observed that the *average number of turn segments* first increased (Fig. 4ii) and then decreased after a peak at 120° (Fig. 4iii) (*Note:* This result is expected because we used the fitting threshold and all curve segments with small steering amplitudes got deleted during pre-processing as part of our procedure). As a trade-off between all three design considerations, we decided to design a process that automatically selects the data of the first quartile after the maximum average number of turn segments (Fig. 4). We then integrated a simple binary classification that contrasted the median of damped natural frequency across that interval between CALM DRIVE and STRESS DRIVE.

In the next step, we applied the automatic turn segmentation process to each individual data. The individual maximum *average number of turn segments* peaks ranged between 70° and 150° *upper bound* across participants. Results revealed that the individualized method correctly detected stress in 77% of the cohort. The process outputs showed the incorrect output for five individuals. All of these five participants had *upper bound* thresholds (i.e., ranging between 70° and 110°) that were lower than the averaged *upper bound* threshold across participants (i.e., 120°).

## Discussion

In this study, we explored whether it is possible to detect stress, expressed through muscle tension in the driver’s arm and shoulder, from steering angle data in an unmodified production car. We developed an LPC-based stress sending method that is (I) *Passive*: measuring non-intrusively, without changes to user behaviour; (II) *Software-based*: no need to retrofit additional sensors, instead we used steering angle data via CAN bus that are available in all modern cars; and (III) *Rapid:* capable of generating an accurate and rapid classification of driver stress (e.g., within 1–2 min of driving) for timely stress management interventions^29,36,37^. We tested the method across cohort and further automated the procedures to detect stress in the individual driver. Results revealed that the method successfully detected stress within the initial eight turns of the drive across the cohort. Further analysis revealed that the automated individualized stress sensing method achieved rapid (i.e., within 8 turns) stress detection in the individual with a detection accuracy of 77%. These results add to the emerging literature of nonintrusive and ubiquitous stress sensing.

Results of cohort analyses demonstrated that the damped natural frequency (as a measure of muscle stiffness and thus stress) was higher during the stress condition compared to the calm condition for the INITIAL 8 TURNS, however, not for the ENTIRE DRIVE period. These findings are partially in contrast to the previous findings in the simulator that showed consistent differences also for the entire drive^33^. The fact that stress cannot be observed through the entire drive (versus the initial eight turns) aligns with our physiological stress observations measured from the ECG data, where we identified a reduction in stress response over time. Results, however, also revealed that the induced stress response upon stress stimulus was higher in this study (*Cohen’s d* = 1.343) compared to the work in the simulator (*Cohen’s d* = 0.633). The combined findings suggest that lower sampling frequency and increased noise in a moving vehicle study did not allow accurate capture of the distress response in participants. Another potential explanation for the missing significance for ENTIRE DRIVE might be the observed difference in LPC-model fit. For ENTIRE DRIVE, the LPC variance of the residual error was higher during CALM DRIVE than STRESS DRIVE, which might have resulted in reduced signal-to-noise ratio. For the INITIAL 8 TURNS, we saw a similar effect size (i.e., *Cohen’s d* = 0.723) compared to the simulator study (i.e., *Cohen’s d* = 0.758). Overall, we can confirm our research hypothesis for the INITIAL 8 TURNS duration.

The results in this study provided further evidence that it is possible to detect stress in the individual. The binary stress sensing classifier reached an accuracy of 77%. Interestingly, all five incorrectly classified participants had AVERAGE NUMBER OF TURN SEGMENTS-peaks that were smaller than the average peak at 120° *upper bound* across participants. This finding indicates that our method requires further engineering to account for different driving speeds and steering behaviour which will potentially increase the classifier accuracy. An overall strategy could be to engineer the sensing method to increase sensitivity (as opposed to specificity) and favor the reduction of false negatives over the reduction of false positives. Nonetheless, with an accurate stress assessment of 77% our study results provide the first proof-of-concept towards stress sensing in the individual. Beyond the collection of the daily stress biomarker, this rapid sensing (i.e., 8 turns) could further allow for just-in-time stress management interventions, e.g., at the start of commute time after a taxing day in the office. This advantage may also be a limitation given that the stress sensing method is inherently limited to driving areas with turns.

A major strength of this study is that we tapped into CAN bus data and did not attach any supplementary sensors. A potential implementation of our method would require only a software upgrade by the car manufacturer, and no mechanical retrofitting of the vehicle. In addition, the stress sensing method does not require any specific human behaviour change, such as using both hands while driving, or consistently driving in the same body position or system calibration (our solution is passive). We did not provide any instructions to the participants that would change their natural driving behaviour, and only invited frequent commuters who are experienced drivers. However, it is important to note that as we move forward towards naturalistic experiments in peoples’ own cars, familiarity with the vehicle may be a factor that needs to be considered, as this may affect the way people handle the steering wheel.

We should note three limitations of this study. First, the sampling size of this study is *N* = 24, although based on the power analysis on the prior simulator data^33^, we estimated a sampling size of *N* = 29 (with *α* = 0.05 and power = 0.8). We were only able to secure exclusive use of the underground parking garage on the Stanford University campus for a limited period of time, and 24 was the maximum number of participants we could accommodate for the test run. Second, to create comparable data between participants and to ensure safety, it was beneficial to run the study in a parking garage before moving into real traffic. Though we mapped the track for eight turns, each of a different radius and approach speed, the parking garage environment prohibited testing the stress sensing method with higher speeds (e.g., as it might be the case at large traffic intersections). A third limitation is the level of ecological validity of the stress stimulus. Because the math stressor was relatively short (i.e., three minutes of length), real-life mental stress was approximated. Absolute stress between calm and stress increased by *M* = 4.09 points, out of 10 (*SD* = 2.58), and two participants did not report increases in perceived stress. Thus, the stress stimulus induced only a moderate stress response. Inducing stress within an experimental context is a major limitation of stress research in general, for us even more so given the dynamic environment of a moving car. At the same time, inducing stress during a driving experiment has ethical limits and includes the risk of accidents. We selected the particular stress stimulus because the TSST math stressor is widely used in other experimental environments^40^ and prior research in a moving vehicle graded this math stressor as safe^37^.

Future studies should test the stress sensing method under realistic conditions, that is, on-the-road with varying speeds, turn radii, and traffic conditions. Other inverse filtering techniques, along with more advanced algorithms and machine learning approaches can potentially increase the binary stress assessment accuracy and allow an extension of the method to real-time stress assessments. Additional engineering research should explore how much more we can decrease the number of turns and still get reliable output. At large, we envision a context-aware, personalized system that can sense a driver’s stress state to apply health enhancing interventions. It will be therefore important to collect longitudinal data of individuals. Potential biases, such as muscle fatigue and natural changes over the day (e.g., morning versus evening commute), must be accounted for.

In conclusion, this study demonstrates that stress can be inferred via steering angle data obtained from a regular production car within the first eight turns of a drive, allowing for a software-only implementation. We extended prior work in the simulator showing that stress, expressed through muscle tension in the arm and shoulder, can be estimated from angular movement data even under the effects of noise while driving. Further engineering of the individualized stress detection method presented here could potentially allow timely monitoring of daily stress biomarkers along with just-in-time stress management interventions while commuting.

## Methods

### Steering wheel stress sensing method

#### LPC-based modelling

The movement of a steering wheel is a rapid, goal-directed, and skilled movement. Research in biomechanics has shown that a rapid goal-directed movement can be modelled as a step response of a linear second order system, i.e., an MSD system^41–45^. Following previous work^30,31^, we make an assumption that the movement of the arm in a steering task can be also modelled as an MSD system. In this context, the *mass* element represents an aggregate sum of the mass of the arm, hand, and steering wheel (Fig. 1). The *spring* element represents the stiffness provided the muscles’ tension in the arm, while the *damper* element represents the total friction in the system of the arm and the steering wheel. It can then be postulated that the input force *F*(*t*) of the arm produces an output movement *X*(*t*) in the steering wheel. The MSD responds with an oscillation frequency driven by the spring component (*k*) and a characteristic decay function determined by the friction of the damper component (*c*). This *damped oscillatory behaviour* can be fully characterized by the damped natural frequency (*ω*) and the *damping ratio *(*ζ*). For a system with constant mass, the damped natural frequency is proportional to the spring coefficient: $\omega \propto \sqrt{k}$.

**Figure 5:**
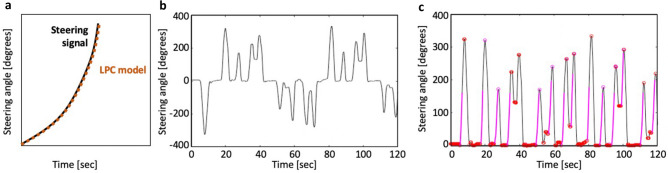
Steering wheel stress sensing method. (**a**) A typical steering wheel movement and corresponding LPC trajectory. (**b**) Steering angle data for one participant with negative values for counter clockwise rotation; and, (**c**) processed steering angle data along with the selected turn segments.

In Fig. 5a, we present a typical steering wheel movement over time. Since we only have the output signal of this MSD system (i.e., movement *X*(*t*) in the steering wheel), we require a model that would allow us to infer the system’s properties, and the damped natural frequency in particular. The motivation is further to implement an inference model that requires minor computational resources. For this purpose, we apply an inverse filtering technique named linear predictive coding (LPC)^32^. LPC builds a predictive model of future samples based only on linear combinations of observed signals from the past^32^. In other words, LPC allows reconstruction of a shape of curve that best fits a given steering wheel signal. The crux is that we can use a linear second-order LPC model, similar to the MSD system, in which cases their coefficients are directly transposable within the Laplace domain^46^. If we build a second-order LPC model that best fits a series of samples, we can recover the MSD parameters. Recent work has shown that theoretical MSD parameters and LPC-inferred parameters correlate strongly using computer mouse movement data^30^. For a mathematical introduction to the proposed LPC-based inference method, we refer the reader to Kim et al.^30^. In the present study, we apply an LPC model using an interpolation order of (*p* = 4) to obtain a sequence of coefficients that can effectively model the underdamped MSD system. The complex roots (r) of this polynomial characterize the MSD’s behaviour. The absolute value of the imaginary part represents the damped natural frequency (ω =|ℑ(r)|), while the ratio of the real part to its absolute value represents the damping ratio ($\zeta =\frac{\left|\mathfrak{R}(r)\right|}{\Vert r\Vert }$). This damped natural frequency and damping ratio enable the inference of muscle stiffness and, hence, stress.

#### Angular data processing

As a driver makes a turn in our study, the steering wheel angle movement is captured at 100 Hz, with a resolution of 0.1° and maximum absolute degree of freedom of 450° in each direction. Prior to applying the LPC transformation, we pre-processed the signal by: (1) eliminating repeated time stamps; (2) transforming steering wheel data from a + (right)/ − (left) representation into its absolute values (0°–450°) (Fig. 5b); and (3) selecting only monotonically increasing segments between valleys (local minima) and peaks (local maxima). In contrast to prior work in the stimulator that chose turn segmentation parameters based on theoretical assumptions^33^, for this study, we chose an empirical approach (i.e., video coding) for our initial cohort analysis (RQ1). Subsequently, we developed a data-driven process that identified the segmentation parameters automatically (RQ2).

#### Empirical turn segmentation process

For our cohort proof-of-concept analysis, we manually selected the turn segmentation parameters based on video coding. Specifically, we inspected time-synched video data of the steering behaviour along with steering angle CAN bus data over a randomly selected two-minute driving segment for each participant. To capture the activation of large muscle groups in arm and shoulder, mostly affected by stress, first we eliminated turn segments that are smaller than 40° (*lower bound)*. We then analysed time-synchronized video of the steering behaviour along with steering angle data over a randomly selected two-minute driving segment for each participant. Video analysis revealed a second steering impulse when a drivers’ initial steering input was followed by a change of grip. We determined the steering angle for starting this second-impulse (*upper bound*) threshold to be at (*M* = 150.2°, *SD* = 23.2) across participants. For turns with steering angles larger than 150°, we cut the curve at this *upper bound* threshold. Figure 5c shows example data of the resulting TRUNCATED TURN SEGMENTS for one participant. In our cohort analysis, we apply LPC to each of the selected TRUNCATED TURN SEGMENTS to derive the segments’ damped frequencies.

#### Automatic turn segmentation process

Angular steering wheel movement will change with driving environment and driving behaviour. To operationalize a real-world implementation of our stress sensing method, it is necessary to automate the process of selecting turn segments that will serve as input to the LPC-based stress sensing method. We chose a data-driven approach to develop the automatic turn segmentation process. Specifically, we used cohort data to define process steps and applied this process to individual data to test the effectiveness of the sensing method. We computed the LPC-MSD parameters for various *upper bound* values changing in increments of 10° until the maximum steering angle was reached. From the resulting *upper bound* vector (all segments versus all participants), we defined processing steps that considered (a) accuracy of fit, approximated by LPC residual error; (b) number of TRUNCATED TURN SEGMENTS; and (c) the sensitivity of the sensor towards variance in damped natural frequency interval values. The process steps are described in the “Results” section.

### Main study

#### Participants

This study was approved by the Stanford University Institutional Review Board and the experiment was performed in accordance with relevant guidelines and regulations. Informed consent was obtained for all participants. We recruited via social media and email. Participants were provided auto-insurance for the duration of the experiment after providing proof of a valid driver’s license. We recruited *N* = 24 participants (12 female, 12 male), with age of *M* = 45.3 years (*SD* = 14.5, min = 20, max = 69), and driver’s license possession of *M* = 28.2 years (*SD* = 14.6) for the experiment. We selected participants to closely represent the population of American commuters (*M* = 42.4 years, *SD* = 14.4)^18^. All participants had normal/corrected to-normal vision and hearing, and no participant had any neck, arm, or shoulder injury during the past 6 months.

#### Experimental protocol

**Figure 6:**
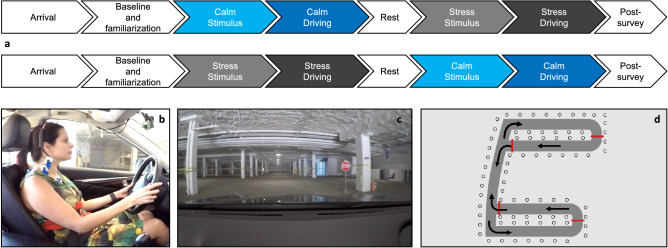
Experimental methodology. Procedure of the experiment (**a**), in which frequent commuters drove in a regular car (**b**), within a large parking garage (**c**), on a fixed 0.65 miles driving course that included eight turns per lap (**d**).

The study procedure consisted of four phases (Fig. 6a): (1) PHYSIOLOGICAL BASELINE and DRIVING FAMILIARIZATION, (2) a STRESS STIMULUS followed by a driving under stress (STRESS DRIVING) period, (3) wash out (REST) period and (4) a CALM STIMULUS followed by a CALM DRIVING period*.* Phases 2 and 4 were counterbalanced to avoid ordering effects. During PHYSIOLOGICAL BASELINE, participants watched a calming video showing a beach scenario for a duration of 3 min. Thereafter, participants drove ten laps on the driving track to complete DRIVING FAMILIARIZATION, which aimed to reduce learning and adaptation effects. The STRESS STIMULUS lasted for three minutes. The stressor was a modified version of the Trier Social Stress Test^47^, in which for three minutes, participants had to calculate backwards from 1521, in steps of 13. If a wrong answer was given or if an answer was not provided within four seconds, which ever came first, the participant had to start over. The STRESS STIMULUS lasted for three minutes. Thereafter, the participants begun the STRESS DRIVING period, in which they drove ten laps while heavy metal music was played to maintain the higher levels of stress^33,48^—the song “At the Heart of Winter” by Immortal https://www.youtube.com/watch?v=VeOlPQqJR-o) was played. After three minutes of washout REST period, participants watched a calming beach scenario video for three minutes (CALM STIMULUS), before driving for ten laps during the CALM DRIVING period listening to the participant’s favourite relaxing music titles or genre (collected during recruitment). All participants stated to dislike heavy metal music in the post-study questionnaire.

#### Apparatus and driving course

We used an unmodified 14-in. diameter steering wheel on a 2016 Infinity Q50 sedan (Fig. 6b). We conducted the experiment in an underground parking garage secured exclusively for this experiment (Fig. 6c,d). We marked out a 10 ft. wide driving course with masking tape to simulate a typical city road^49^. The driving course was 0.65 miles long and included four left and four right turns of varying radii, and four intersection stop signs, making all eight turns different from one another (Fig. 6d). Each participant drove 10 laps, covering a total of 6.5 miles and 80 turns. The experimenter instructed the participants to imagine driving in the city and did not specify a speed limit. Importantly, we refrained from providing any driving instructions and gave the drivers the discretion to adjust their seating positions and steering manner (i.e., one-arm versus two-arm driving).

#### Driving data

We collected steering wheel data via the controller area network (CAN) bus, which is a standard protocol used in the on-board diagnostic (OBD-II) for all cars and light trucks sold in the United States since 1996^50^. Thus, our approach could potentially be translated to any other car manufactured after that year. Steering wheel data was sampled at 100 Hz, with a resolution of 0.1° and maximum angle of 450° in each direction. Positive angle rotations are recorded when the wheel turns clockwise, i.e., when the car turns right, and negative angle rotations are recorded when the wheel turn counter clockwise, i.e., when the car turns left. The data were noticeably inferior in quality to the 11-inch diameter Logitech G29 Gaming Steering Wheel^51^ data with a 916 Hz sampling rate and an angular resolution of 0.056° from the simulator pilot study^33^. Additionally, we collected ancillary driving behaviour data such as speed and acceleration. Finally, three cameras captured the frontal and side views of the participant, and the frontal view of the road.

#### Psychophysiological data

Participants wore a Zephyr Bioharness 3.0^52^ to record ECG data (at 250 Hz), to derive *heart rate* and *heart rate variability*. To derive HR and HRV from the ECG signal, we used Kubios 2.2 HRV software 2.2^53^. We excluded one participant due to corrupted ECG data, likely because the sensor belt was not fastened properly and therefore induced noise. We first ran the automatic peak detection, and then visually verified the results to remove false positives and false negatives and extracted the corresponding mean HR (bpm) and HRV-RMMSD (msec) values.

#### Self-report data

Self-reported data was collected five times (once after each phase of the study procedure). Self-reported stress (SRS) was obtained from a simplified version of the Perceived Stress Scale^54^ (“How stressed do you feel right now?”) with 10-point scale ranging from “0-low” to “10-high”. Additionally, we collected level of *arousal* (“How energized do you feel right now?”) with an adapted 10-point scale ranging from “0-sleepy” to “10-energized”; and level of *valence* (“How pleasant do you feel right now?”) with an adapted 10-point scale from “0-unpleasant” to “10-pleasant”—derived from the Affect Grid^55^. As an ancillary measure we collected the level of *physical tension* (“How physically tense do you feel right now?”) with a 10-point scale ranging from “0-low” to “10-high”.

## Supplementary Information

Supplementary Information.
