# Supplementary material for: Individualized stress detection using an unmodified car steering wheel
Source: Sci Rep. 2021 Oct 19;11:20646. doi: 10.1038/s41598-021-00062-7 (PMC8526569; doi:10.1038/s41598-021-00062-7)
Supplement: Supplementary file 1 — Supplementary Information. [file 41598_2021_62_MOESM1_ESM.docx]

## Supplementary Material

## Individualized Stress Detection using an Unmodified Car Steering Wheel


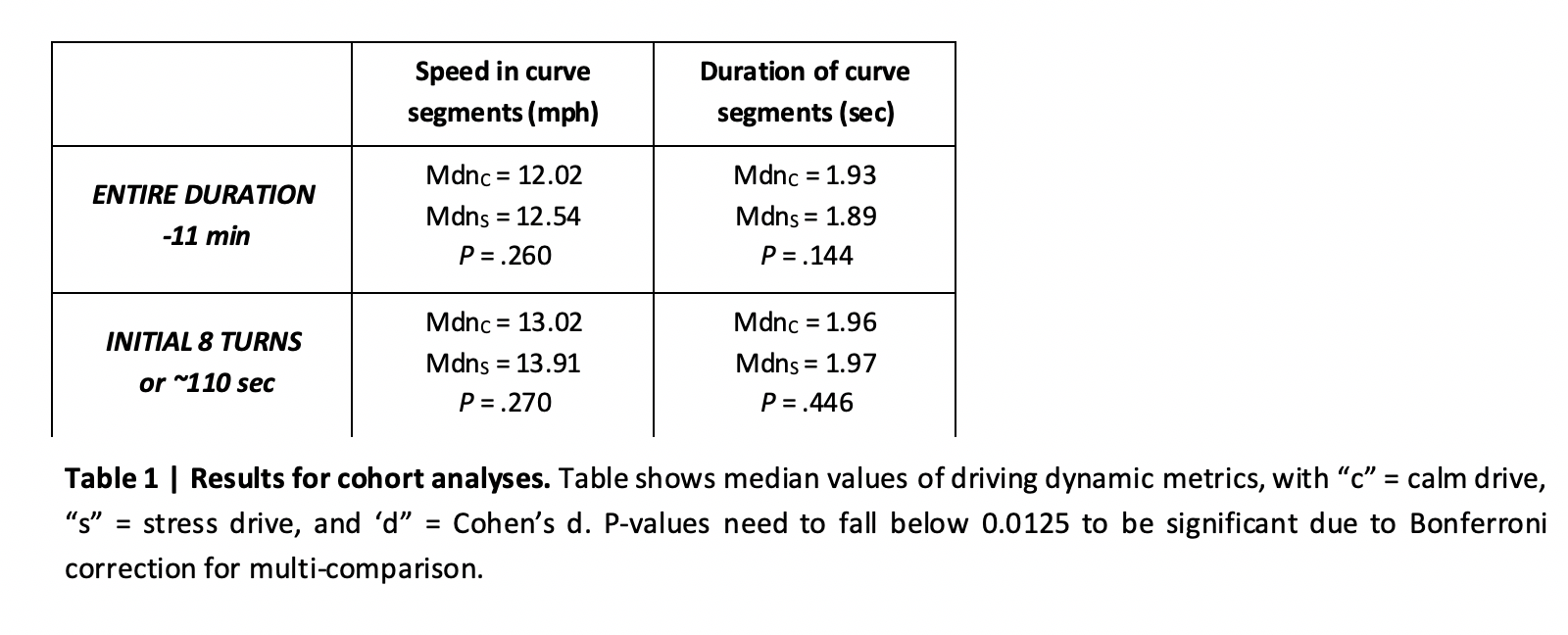

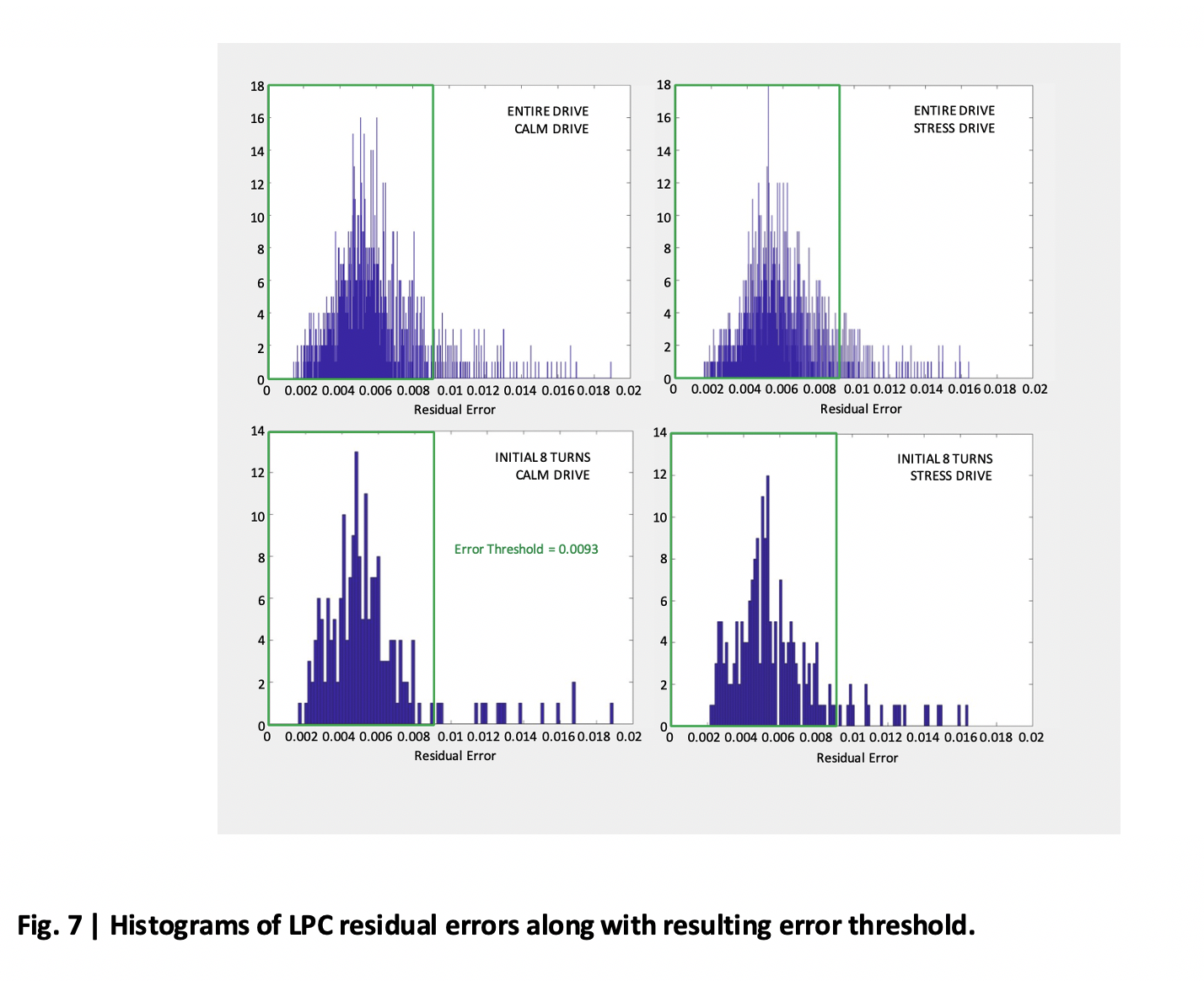


**Fig. S1 | Histograms of LPC residual errors along with resulting error threshold.**
